# Supplementary material for: Altered microRNA Transcriptome in Cultured Human Liver Cells upon Infection with Ebola Virus
Source: Int J Mol Sci. 2021 Apr 6;22(7):3792. doi: 10.3390/ijms22073792 (PMC8038836; doi:10.3390/ijms22073792)
Supplement: Supplementary file 1 [file ijms-22-03792-s001.zip › Supplementary_File/C_ GO_Analysis_Results/16-30nt_go_Mayinga-96h-Huh7_vs_Control-96h-Huh7_down.mature_mirna_targets/MF_FoldEnrichmentDotPlot.pdf]

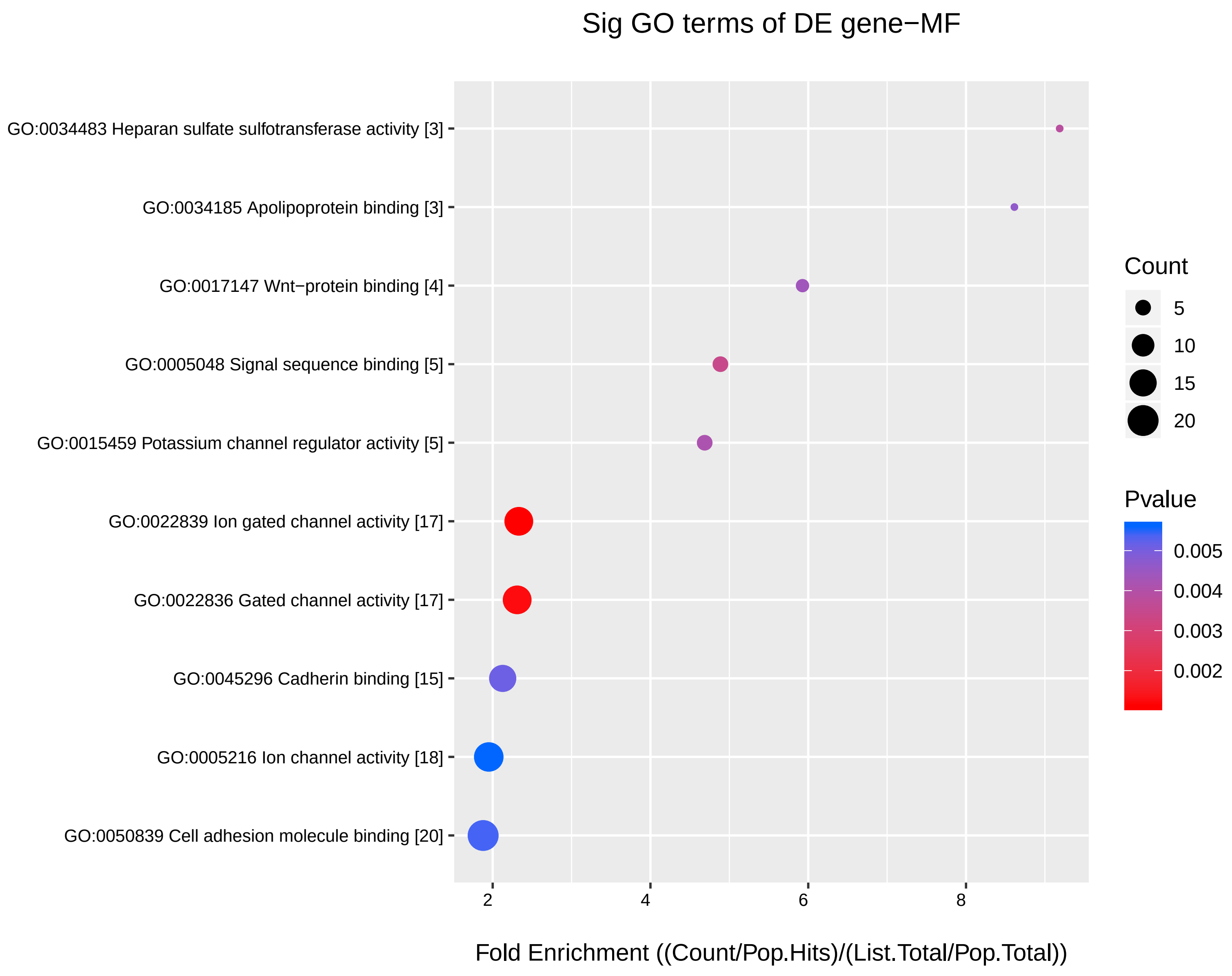
Sig GO terms of DE gene−MF
GO:0034483 Heparan sulfate sulfotransferase activity [3]
GO:0034185 Apolipoprotein binding [3]
GO:0017147 Wnt−protein binding [4]
GO:0005048 Signal sequence binding [5]
GO:0015459 Potassium channel regulator activity [5]
GO:0022839 Ion gated channel activity [17]
GO:0022836 Gated channel activity [17]
GO:0045296 Cadherin binding [15]
GO:0005216 Ion channel activity [18]
GO:0050839 Cell adhesion molecule binding [20]
2
4
6
8
Fold Enrichment ((Count/Pop.Hits)/(List.Total/Pop.Total))
Count
5
10
15
20
Pvalue
0.005
0.004
0.003
0.002
